# Mitochondrial respiratory supercomplexes associated with longevity in mammals

**DOI:** 10.3389/fragi.2026.1876149

**Published:** 2026-07-29

**Authors:** Shinichiro Suzuki, Kazuhiro Ikeda, Toshihiko Takeiwa, Kuniko Horie, Satoshi Inoue

**Affiliations:** 1 Division of Systems Medicine and Gene Therapy, Faculty of Medicine, Saitama Medical University, Saitama, Japan; 2 Department of Systems Aging Science and Medicine, Tokyo Metropolitan Institute for Geriatrics and Gerontology, Tokyo, Japan

**Keywords:** lifespan, longevity, mitochondria, OXPHOS, supercomplex

## Abstract

Because of population aging and morbidity expansion, extending healthspan has become a global challenge and it is required to elucidate molecular mechanisms underlying aging and age-related diseases. Mitochondrial dysfunction is a hallmark of aging, characterized by impaired oxidative phosphorylation, increased production of reactive oxygen species (ROS), and metabolic imbalance. Therefore, maintaining mitochondrial homeostasis is essential for healthspan. Mitochondrial respiratory chain complexes organize into higher-order assemblies known as supercomplexes (SCs), which enable to efficient energy or ATP production with repressed ROS generation. Notably, the assembly and stability of these SCs likely decline in aged mammals. In addition, factors such as COX7RP/SCAF1 and mitochondrial lipid cardiolipin have emerged as key regulators of SC assembly. In this review, we summarize the molecular assembly, physiological roles, and longevity implications of SC in healthy mammals. We further discuss emerging evidence supporting SC modulation as a potential strategy for promoting healthy aging.

## 1 Introduction

The global population is rapidly aging, as the population aged 65 or over is currently approximately 830 million people and it will reach 2.2 billion by 2070 based on World Population Prospect 2024. Furthermore, the healthspan-lifespan gap is globally widening, from 8.5 years in 2000 to 9.6 years in 2019 (Garmany and Terzic, 2024). These trends underscore the importance of extending healthspan and compressing morbidity. Therefore, understanding the biological mechanisms of aging and developing effective interventions are urgent priorities.

One of the hallmarks of aging is mitochondrial dysfunction (Lopez-Otin et al., 2023). Mitochondria are multifunctional organelles, a central function of which is the generation of cellular energy ATP through oxidative phosphorylation (OXPHOS) (Kuhlbrandt, 2015). The OXPHOS system consists of five complexes I-V, with complexes I-IV forming the electron transport chain that transfers electrons from NADH and FADH_2_ to oxygen while generating a proton gradient across the inner mitochondrial membrane (IMM). This gradient drives ATP synthesis by complex V.

The abundance and activity of OXPHOS components likely decline during aging, such as reduced levels and activity of complex I, and a declining trend in complexes III and IV (Emelyanova et al., 2018). Consistently, animal models with OXPHOS defects exhibit shorter lifespans than wild type controls (Trifunovic et al., 2004; Kujoth et al., 2005). Age-associated deterioration of mitochondrial OXPHOS is assumed to arise through multiple mechanisms, including the accumulation of mitochondrial DNA (mtDNA) mutations, which contributes to increased ROS production and the promotion of cellular damage (Salmonowicz and Szczepanowska, 2025). Therefore, mitochondrial integrity including OXPHOS is considered a critical component of healthy lifespan extension.

**FIGURE 1:**
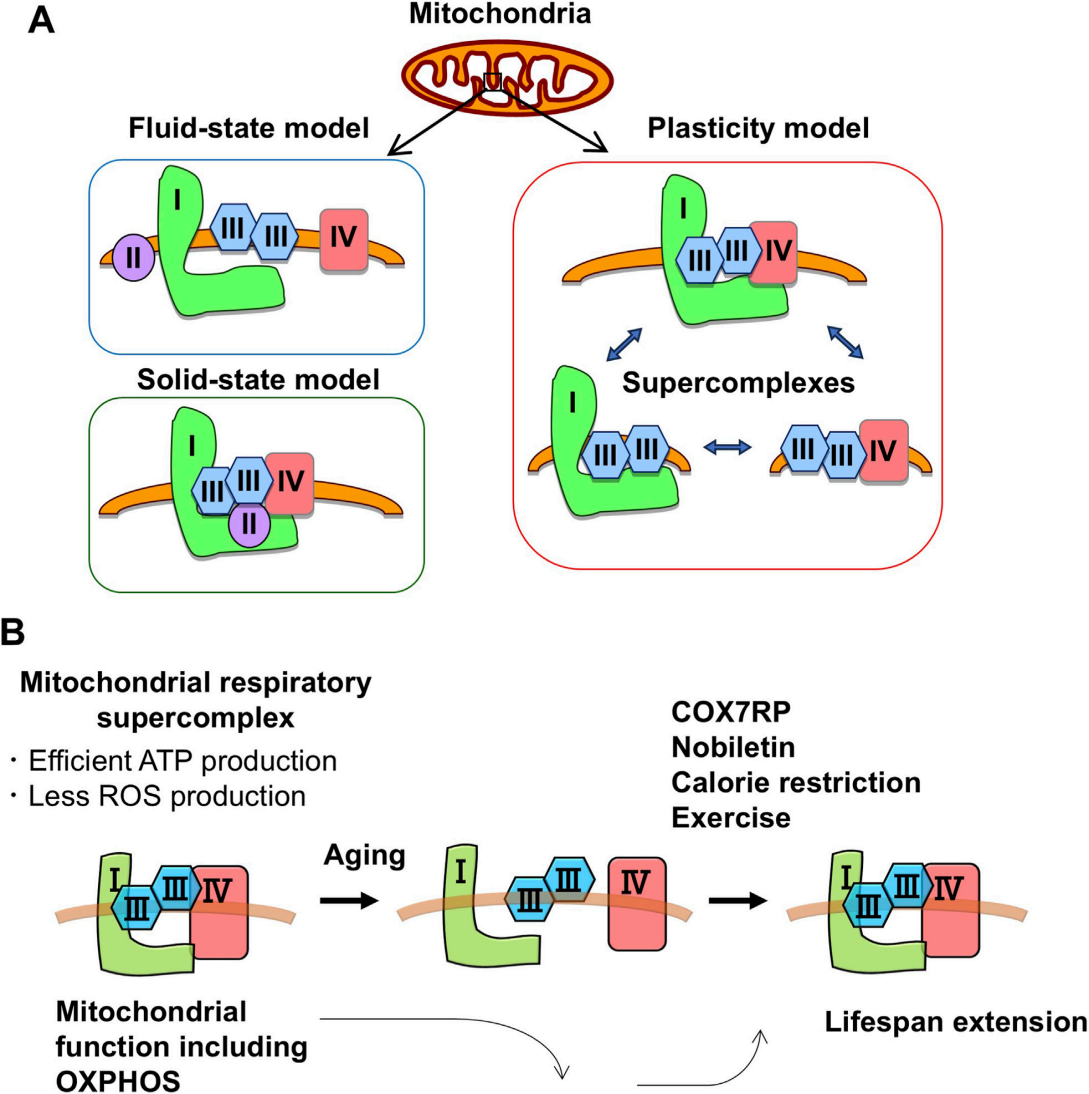
Mitochondrial respiratory supercomplexes extend lifespan. **(A)** Models of mitochondorial respiratory chain organization. The organization of mitochondrial respiratory chain complexes is described by three major models: the fluid-state model, the solid-state model, and the plasticity model, which integrates features of both free complexes and supercomplexes. I, II, III, and IV denote mitochondrial respiratory chain complexes I, II, III, and IV, respectively. **(B)** COX7RP, nobiletin, calorie restriction, and exercise enhance the formation of the supercomplexes while aging decrease the formation. Previous reports suggest the possibility that the formation of supercomplexes is involved in extend lifespan. COX7RP, cytochrome *c* oxidase subunit 7a related polypeptide; ROS, reactive oxygen species; OXPHOS, oxidative phosphorylation.

Regarding the organization of OXPHOS components within the IMM, two classical models have been proposed (Figure 1A). The fluid-state model posits that respiratory chain complexes and mobile electron carriers independently diffuse within the IMM, and transfer electrons through random collisions, whereas the solid-state model proposes that complexes I, III, and IV assemble into stable supercomplexes that facilitate electron transfer. More recently, the plasticity model has been proposed, in which complexes I, III, and IV of the electron transport chain can assemble into SCs, forming higher-order structures (Schägger and Pfeiffer, 2000; Vonck and Schäfer, 2009). The physiological significance of OXPHOS supercomplexes, including their proposed role in enhancing ATP production efficiency, remains a matter of ongoing debate (Nath, 2022). However, we propose that SCs play an important role in promoting ATP production and suppressing ROS generation (Ikeda et al., 2013; 2019; 2026). In this review, we focus on the function of SCs and their contribution to longevity in mammals and discuss potential interventions that could extend healthspan.

## 2 Assembly and role of SCs in OXPHOS

Blue native-polyacrylamide gel electrophoresis and cryo-electron microscopy have led to a paradigm shift in the field of SC research, revealing their precise architecture, stability, and functional mechanisms (Schägger and Pfeiffer, 2000; Dudkina et al., 2010; Guan et al., 2022) (Figure 1A). These techniques have shown that respiratory complexes (I, III, and IV) form larger complexes or SCs. Complexes I, III, and IV form various SCs, including I + III_2_, I + III_2_+IV_n_ (n = 1–4), and III_2_ + IV_n_ (n = 1 or 2) (Letts et al., 2016). Among them, the I + III_2_+IV_n_ assembly is referred to as the respirasome, representing a model for a network of respiratory chain complexes (Schägger and Pfeiffer, 2000). As we discuss later, cytochrome *c* oxidase subunit 7a-related polypeptide (COX7RP), or supercomplex assembly factor 1 (SCAF1)/cytochrome *c* oxidase subunit 7a2 like (COX7A2L), has been identified as an SC assembly factor that advanced our understanding of SC organization and function (Ikeda et al., 2013; 2019; Kobayashi et al., 2023; Lapuente-Brun et al., 2013; Schägger et al., 2004; Maranzana et al., 2013).

SCs are thought to enhance mitochondrial function by facilitating electron transfer, improving the efficiency of ATP production, modulating the assembly and stability of complexes I and IV, and limiting electron leakage that leads to ROS generation. However, the mechanistic basis of these effects remains a matter of ongoing debate (Guan et al., 2022; Vercellino and Sazanov, 2022; Lenaz et al., 2024; Lapuente-Brun et al., 2013; Fedor and Hirst, 2018). In particular, whether SCs enable substrate channeling of electron carriers such as coenzyme Q (CoQ), thereby creating functionally insulated CoQ pools, or instead operate through a shared, freely diffusible CoQ pool, is still controversial. Recent studies suggest that, rather than forming rigid electron-conducting units, SCs may act as dynamic assemblies that modulate electron flux and redox balance in response to metabolic conditions (Guan et al., 2022), highlighting their role in mitochondrial plasticity while leaving key aspects of their functional organization unresolved.

The relationship between ROS and SCs appears to be complex and dynamically regulated. Recent studies have demonstrated that ROS-mediated oxidation of cysteine residues within NDUFS1, a core subunit of complex I, promotes SC assembly, thereby reducing electron leakage and limiting further ROS production (Chen et al., 2025). This finding suggests the existence of a physiological feedback mechanism that contributes to mitochondrial redox homeostasis. However, such reversible adaptive responses may become impaired during aging, leading to persistent oxidative damage and mitochondrial dysfunction. One potential mechanism underlying the age-related attenuation of SC-mediated adaptation to oxidative stress involves cardiolipin. Cardiolipin is an essential phospholipid of the IMM that stabilizes respiratory chain complexes and promotes SC assembly. Notably, cardiolipin is highly susceptible to oxidative damage, and oxidative stress-induced cardiolipin oxidation has been shown to trigger its degradation (Liu et al., 2022). Therefore, the accumulation of oxidative stress during aging may impair SC stability through cardiolipin oxidation, thereby diminishing the compensatory increase in SC assembly in response to ROS.

## 3 Reduction of SCs with aging

Several studies have demonstrated an age-associated decline in SC assembly (Figure 1B; Table 1). In the heart tissue from male Fischer 344 rats, 24-month-old animals exhibited approximately 15% lower levels of SCs than 5-month-old counterparts (Gomez et al., 2009). Soleus muscle tissues from 26-month-old female Sprague-Dawley rats contained less than half the amount of SCs detected in 8-month-old animals (Han et al., 2022). In the cerebral cortex of male Wistar rats, the levels of III_2_ + IV, I + III_2_, I + III_2_+IV, I + III_2_+IV_2_, and I + III_2_ + IV_3_ were reduced by 1.5%, 58.0%, 30.8%, 40.7%, and 13.8%, respectively, in 30-month-old rats compared with 5-month-old rats (Frenzel et al., 2010). Mitochondria isolated from the gastrocnemius muscle of male Wistar rats showed a reduction in the lighter SCs, in particular decrease in I + III_2_ levels by approximately 40%, at 24 months relative to 3 months of age, while the heavier SCs increased, possibly reflecting a compensatory response (Lombardi et al., 2009). Both male and female C57BL/6 mice exhibited lower levels of I + III_2_ in the brain at 26 months of age than at 6 months (Liang et al., 2025). In contrast, naked mole-rats, a species notable for exceptional longevity and resistance to senescence, exhibit age-associated increases in I + III_2_ and I + III_2_ + IV_n_, implying that the maintenance or enhancement of the SC abundance may contribute to resistance to aging. These observations suggest that tissue-specific differences in SC dynamics may contribute to variations in mitochondrial resilience during aging, although it remains to be elucidated. Nevertheless, one possible explanation is that reduced expression of respiratory chain complex subunits, regulatory factors involved in their assembly, and factors promoting SC formation such as COX7RP, may contribute to a decline in SC formation, although direct evidence remains limited. It is also possible to speculate that the accumulation of mitochondrial damage (e.g., mtDNA mutations and lipid peroxidation) may indirectly impair the SC assembly.

**TABLE 1:** Longevity- and lifespan extension-associated alterations in mitochondrial respiratory supercomplex assembly under normal physiological conditions in mammals.

Animal model/treatment	Tissues	Sex	Age	Comparison	Supercomplexes	Alteration	References
Wild-type animals							
Fischer 344 rats	Heart	Male	24 months old	5 months old	Total SCs, S_4_, S_3_, and S_2_	decrease	Gomez et al. (2009)
Sprague-Dawley rats	Soleus muscle	Female	26 months old	8 months old	SC	decrease	Han et al. (2022)
Wistar rats	Cortex	Male	30 months old	5 months old	I + III_2_, I + III_2_ + IV, and I + III_2_ + IV_2_	decrease	Frenzel et al. (2010)
Wistar rats	Gastrocnemius muscle	Male	24 months old	3 months old	lighter SCs	decrease	Lombardi et al. (2009)
					heavier SCs	increase	
Naked mole-rats	Brain	Female	15.38 and 15.94 years old	2.77 and 4.57 years old	I + III_2_ and I + III_2_ + IV_n_	increase	Liang et al. (2025)
		Male	32.70 years old	2.77 years old	I + III_2_ and I + III_2_ + IV_n_	increase	
C57BL/6 mice	Brain	Female	26 months old	6 months old	I + III_2_	decrease	Liang et al. (2025)
		Male	26 months old	6 months old	I + III_2_	decrease	
Treatment/gene modification							
*COX7RP* transgenic mice (C57BL/6)	WAT and quadriceps femoris muscle	Male	2 years old	wild-type	III_2_ + IV and I + III_2_ + IV_n_	increase	Ikeda et al. (2026)
Nobiletin and high-fat diet (C57BL/6)	Skeletal muscle	Male	20- to 22-month-old	high-fat diet	I_n_ + III_n_	increase	Nohara et al. (2019)
Clorie restriction (C57BL/6)^a^	Liver	Male	12 months old	ad libitum	SC	increase	Kim et al. (2015)
Endurance exercise (Human)^a^	Skeletal muscle	Both	60- to 80-year-old	Before exercise	I + III + IV_n_	increase	Greggio et al. (2017)

^a^ Although these papers do not directly analyze life extension, other studies have demonstrated that it has a life-extending effect (Di Francesco et al., 2024; Reimers et al., 2012).

## 4 The function of COX7RP and its role in longevity

COX7RP was originally identified as an estrogen-responsive protein (Watanabe et al., 1998) and was later recognized as a key regulator of SC formation (Ikeda et al., 2013; Lapuente-Brun et al., 2013). COX7RP is highly expressed in high energy-demanding tissues such as the liver, skeletal muscle, and heart (Zhang et al., 2016). Structurally, COX7RP can form salt bridges with both complex III (Cogliati et al., 2016) and complex I (Letts et al., 2016). The assembly of the III_2_+IV SC from the III_2_ precursor proceeds through a conformational transition driven by COX7RP, in which its N-terminus inserts deeply into complex III_2_, while its C-terminus is incorporated into complex IV (Vercellino and Sazanov, 2021). In addition, the His73 residue of COX7RP is presumed to play a critical role in interactions with other complex IV subunits (Cogliati et al., 2016).

Functional analyses further highlight the physiological importance of COX7RP (Ikeda et al., 2013; 2026; Shiba et al., 2017). In *Cox7rp*-deficient murine embryonic fibroblasts (MEFs), oxygen consumption and ATP production are reduced (Ikeda et al., 2013). *Cox7rp* knockout mice exhibit reduced COX activity and exercise tolerance, impaired thermogenesis, and altered glucose metabolism (Ikeda et al., 2026; Shiba et al., 2017). COX7RP also contributes to metabolic adaptation under hypoxic conditions. In MCF-7 cells, COX7RP overexpression confers hypoxia tolerance, associated with enhanced SC assembly, increased oxygen consumption, and reduced ROS production even under hypoxia (Ikeda et al., 2019). Metabolomic analyses further indicate that COX7RP regulates the steady-state levels of tricarboxylic acid (TCA) cycle intermediates, likely through glutamine-dependent production of succinate and malate (Ikeda et al., 2019). The role of COX7RP in modulating mitochondrial respiration and metabolic pathways underscores the importance of SC organization in maintaining cellular energy homeostasis.

Transgenic mice overexpressing *COX7RP* exhibited a significant extension of lifespan with multiple physiological improvements (Ikeda et al., 2026). Notably, *COX7RP* transgenic (*COX7RP*-Tg) mice displayed improved metabolic homeostasis, including enhanced insulin sensitivity, glucose regulation, and a favorable lipid profile with reduced circulating triglyceride and total cholesterol levels. The mice displayed enhanced exercise endurance and reduced fat accumulation in the white adipose tissue. In *COX7RP*-Tg white adipose tissue (WAT) and quadriceps muscle, increased formation of SCs was observed, accompanied by elevated ATP production. Several aging-associated phenotypes were ameliorated in *COX7RP*-Tg WAT, including increased nicotinamide adenine dinucleotide (NAD^+^) levels, reduced ROS production, and decreased senescence-associated β-galactosidase (SA-β-gal) activity. Genes associated with age-related inflammatory processes, particularly senescence-associated secretory phenotype (SASP), were downregulated in aged *COX7RP*-Tg mice compared with wild-type controls.

The putative reduction in senescent cells may also be a pivotal mechanism underlying lifespan extension in *COX7RP*-Tg mice. In humans, senescent cells accumulate in multiple tissues in an age-dependent manner, with reported increases ranging from approximately 2- to 20-fold in healthy older individuals over 65 years old compared with younger individuals under 35 years old (Lopez-Otin et al., 2023; Tuttle et al., 2020). Cellular senescence can occur in essentially all cell types during aging. Senescent cells secrete SASP factors that promote chronic inflammation. Consistent with their detrimental effects, the genetic or pharmacological elimination of senescent cells has been shown to improve healthspan and extend lifespan in naturally aged mice (Xu et al., 2018).

## 5 Nobiletin enhances SC assembly to promote healthy aging

Nobiletin, a flavonoid derived from citrus peel, has been reported to exert anti-aging effects (Nohara et al., 2019). In male C57BL/6 mice, dietary supplementation with nobiletin extended lifespan compared with a regular diet alone. Age-related impairments in glucose tolerance, basal body temperature, and cold tolerance were alleviated by nobiletin treatment. In aged mice fed with high-fat diet, nobiletin treatment increased the abundance of the SC I_n_ + III_n_ in skeletal muscle. Nobiletin also improved grip strength and exercise capacity, and reduced body weight, visceral adiposity, and serum levels of free glycerol and triglycerides in aged mice fed with high fat diet.

## 6 Calorie restriction contributes to SC formation and lifespan extension

Calorie restriction (CR) has been proposed as an intervention with anti-aging effects (Lopez-Lluch and Navas, 2016). Female Diversity Outbred (DO) mice subjected to CR from 6 months of age exhibit extended lifespan (Di Francesco et al., 2024). CR increases the abundance of SCs. (Kim et al., 2015). In 12-month-old male C57BL/6 mice, a 30% CR regimen for 12 weeks promoted SC assembly and increased the expression of cytochrome *c* oxidase subunit 6b1 (Cox6b1) in the liver. Overexpression of Cox6b1 in NIH-3T3 cells increased SC abundance. These observations raise the possibility that CR contributes to anti-aging, at least partially, by promoting SC assembly.

## 7 Exercise facilitates SC assembly and extends longevity

Regular physical activity in humans has been associated with an increase in life expectancy ranging from 0.4 to 6.9 years in human (Reimers et al., 2012). In mice, swimming exercise by 4 months old improved healthspan: enhanced systemic metabolism, cardiovascular function, and muscle strength were observed, with reduced systemic inflammation and frailty in aged animals (Feng et al., 2025). In a study of skeletal muscle biopsies from older adults with endurance training, endurance exercise increased the abundance of mitochondrial respiratory complexes in the skeletal muscle compared with sedentary individuals (Greggio et al., 2017). Exercise promoted the redistribution of complexes I, III, and IV into SCs, particularly I + III_2_ + IV_n_, and enhanced mitochondrial respiration in skeletal muscle.

## 8 Cardiolipin and its interactors prohibitins and STOML2 contribute to SC assembly

As abovementioned, cardiolipin is a factor that promotes SC assembly. Cardiolipin constitutes approximately 20% of the total phospholipid content of the IMM (Horvath and Daum, 2013). Compared with other fluid solvent phospholipids like phosphatidylcholine, non-annular lipid cardiolipin facilitates the stabilization of SC assembly interface with high surface ruggedness (Letts et al., 2016), because of its unique chemical structure consisting of a double glycerophosphate backbone and four fatty acyl chains (Ahmadpour et al., 2020). Cardiolipin-binding sites have been identified in respiratory complexes I, III, and IV, and cardiolipin is particularly important for stabilizing SCs containing complexes III and IV (Musatov and Sedlak, 2017; Fiedorczuk et al., 2016; Lange et al., 2001; Zhang et al., 2002). Intriguingly, the treatment of cardiolipin-binding peptide elamipretide enhanced the activity of complex IV-containing SCs in heart tissues from heart failure patients (Chatfield et al., 2019). In aged mice, elamipretide treatment reversed the age-associated decline in maximal mitochondrial ATP production in skeletal muscle and improved exercise tolerance (Campbell et al., 2019).

Mitochondrial proteins prohibitins (PHBs) including PHB1 and PHB2 form a massive ring-like scaffold in the IMM, locally compartmentalizing and concentrating phospholipids such as cardiolipin: thus, the PHBs generate cardiolipin-enriched lipid rafts, leading to the facilitation of SC formation (Osman et al., 2009; Jian et al., 2017). The PHB complex has been implicated in age-related human diseases like Parkinson’s disease (Dutta et al., 2018) and prostate cancer (Gamble et al., 2007).

Stomatin-like protein 2 (STOML2) is another factor localized to the IMM and interacts with the PHB complex to contribute to the formation of cardiolipin-enriched microdomains, maintenance of mitochondrial structural integrity, and OXPHOS assembly (Da Cruz et al., 2008; Mitsopoulos et al., 2015). STOML2 directly binds to NDUFS4 (a Complex I subunit) to tether early assembly intermediates and acts as a crucial structural hub that physically links SC assembly with the maintenance of cristae architecture via the mitochondrial contact site and cristae organizing system (Mise et al., 2024). STOML2 may also function as a critical guardian of mitochondrial structural integrity and proteostasis during aging, as it mitigates age-associated bioenergetic decline (Mitsopoulos et al., 2015) and confers robust neuroprotection against age-related neurodegenerative diseases (Zanon et al., 2017).

PHBs and STOML2 may influence mitochondrial function through their roles in membrane organization and the maintenance of SC. Further elucidation of these mechanisms may provide new opportunities for promoting healthy aging and extending human healthspan.

## 9 Screening of SC assembly-regulating genes/compounds

Lifespan extension has been observed in various genetically modified models and upon pharmacological interventions (Taormina et al., 2019; Moskalev et al., 2022), although their relationship with SCs remains largely unexplored. Notably, some of these models (Brown-Borg et al., 2012; Elmansi and Miller, 2023) exhibit enhanced mitochondrial OXPHOS capacity and metabolic activity.

We recently developed a Förster resonance energy transfer (FRET)-based respirasome assembly assay between complexes I and IV combined with quantitative evaluation of SC formation and identified spleen tyrosine kinase (SYK) inhibitors that promote SC assembly, such as 3,4-methylenedioxy-β-nitrostyrene (MNS) (Kobayashi et al., 2023). In mice, MNS treatment elevated oxygen consumption during a forced treadmill exercise test and improved exercise performance. A chemical screen using a NanoLuciferase complementation reporter to monitor the proximity between complexes III and IV showed that inhibitors of dihydroorotate dehydrogenase (DHODH), a key enzyme in *de novo* pyrimidine biosynthesis, were identified as potent enhancers of SC assembly (Bennett et al., 2021).

## 10 Conclusion

SCs have emerged as critical regulators of mitochondrial function, with important implications for longevity: SC assembly enhances OXPHOS efficiency, stabilizes respiratory chain components, and limits excessive ROS production, thereby contributing to cellular homeostasis. Conversely, aging is associated with a decline in SC abundance.

Despite these advances, questions remain whether SC assembly factors decline during aging, and whether SC disassembly causally contributes to aging phenotypes. Furthermore, the tissue-specific roles of SC organization, as well as the molecular mechanisms governing their dynamic regulation, remain to be elucidated.

In conclusion, SCs represent a promising but incompletely understood target in aging biology. Elucidating their regulatory mechanisms and physiological significance will facilitate developing novel therapeutic strategies for promoting healthy aging and longevity.
